# Supplementary material for: Effects of muscle mass on muscle force predictions in human movement
Source: bioRxiv. 2026 May 2:2026.03.30.714909. Originally published 2026 Apr 2. Preprint. [Version 2] doi: 10.64898/2026.03.30.714909 (PMC13060126; doi:10.64898/2026.03.30.714909)
Supplement: Supplement 1 [file NIHPP2026.03.30.714909v2-supplement-1.pdf]

bioRxiv preprint doi: https://doi.org/10.64898/2026.03.30.714909; this version posted May 2, 2026. The copyright holder for this preprint (which was not certified by peer review) is the author/funder, who has granted bioRxiv a license to display the preprint in perpetuity. It is made available under aCC-BY 4.0 International license.

Supplementary Information

Supplementary Figures

bioRxiv preprint doi: https://doi.org/10.64898/2026.03.30.714909; this version posted May 2, 2026. The copyright holder for this preprint (which was not certified by peer review) is the author/funder, who has granted bioRxiv a license to display the preprint in perpetuity. It is made available under aCC-BY 4.0 International license.

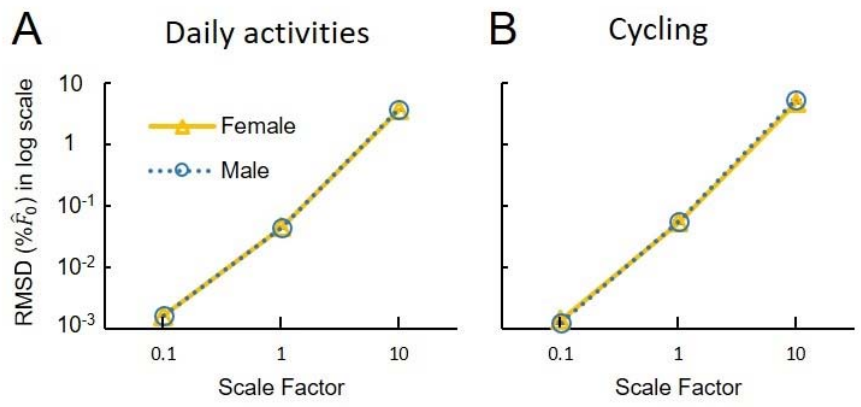

Fig. S1

The effect of sex on differences in model-predicted forces across scaled muscle sizes. Each plotted point denotes the average of log-transformed RMSD values across participants, muscles, and tasks for a specific scale and sex. The standard error associated with each mean value is small enough to be encompassed by the mean's data point. The RMSD values of both sexes overlap nearly completely. ANOVA showed no significant effect of sex on the log-transformed RMSD values across scales.

bioRxiv preprint doi: https://doi.org/10.64898/2026.03.30.714909; this version posted May 2, 2026. The copyright holder for this preprint (which was not certified by peer review) is the author/funder, who has granted bioRxiv a license to display the preprint in perpetuity. It is made available under aCC-BY 4.0 International license.

Supplementary Tables

Participant	Age (y)	Weight (kg)	Height (m)	Sex	Ethnicity
1	25	54	1.75	F	White
2	41	66	1.67	M	Asian
3	32	56	1.56	F	White
4	22	84	1.86	M	Asian
5	26	72	1.65	F	Asian
6	23	77	1.78	M	Middle East
7	24	47	1.55	F	Asian
8	24	58	1.63	F	Asian
9	27	62	1.77	F	Asian
10	40	72	1.73	M	White
11	32	72	1.71	M	White
12	27	65	1.63	F	Asian
13	33	79	1.73	M	Asian
14	41	74	1.73	M	Asian
15	41	75	1.93	M	White
16	22	77	1.83	M	Asian
17	67	68	1.67	M	Asian
18	40	57	1.6	F	Asian
19	40	57	1.63	F	Asian
20	33	56	1.61	F	Asian

Table S1. Characteristics of the 20 participants tested.

To be included in the study, participants must be adults with normal lower limb function who can independently perform walking, running, hopping, and sit-to-stand movements. People with lower extremity musculoskeletal injuries, neuromuscular diseases, or other systemic diseases affecting their ability to perform the required tasks were excluded.
